# Are Certain Foods Addictive?

**DOI:** 10.3389/fpsyt.2014.00038

**Published:** 2014-04-07

**Authors:** Adrian Meule

**Affiliations:** ^1^Department of Psychology I, Institute of Psychology, University of Würzburg, Würzburg, Germany; ^2^Hospital for Child and Adolescent Psychiatry, LWL University Hospital of the Ruhr-University Bochum, Hamm, Germany

**Keywords:** food addiction, Yale Food Addiction Scale, obesity, binge eating disorder, bulimia nervosa

In a recent article (1), Dr. Rippe highlights that lifestyle medicine practitioners need to ground their recommendations on sound scientific evidence and that this is complicated by the fact that scientific information is often distorted and conjecture is sometimes confused with proof. This includes, for example, that associations between variables found in cross-sectional studies are presented as causal relationships or that associations between variables found in epidemiological studies are often confounded by important third variables.

The author illustrates several examples of findings that are often misinterpreted and presented as true facts, although existing evidence needs to be critically evaluated. Those include the notions that (a) sugar causes obesity, (b) certain foods are addictive, (c) certain foods cause cancer, (d) exercise is not effective for weight loss, and that (e) there is a causal link between sugar consumption and diabetes.

I think that the author makes an important point in arguing that scientific evidence often is distorted by researchers or the media and that researchers and practitioners in the field of health behaviors continuously need to take care of scrutinizing research findings. Although I agree with most of the statements made in that article, I also think that some of them on the current concept of food addiction warrant a more in-depth discussion.

## Appropriateness of Animal Models

Firstly, it is stated that “much of the argument related to food and addiction is based on […] animal data” and that those models “may be poorly mimicked in human beings when it comes to food consumption.” Presumably, the author refers to paradigms that show addiction-like consumption of sugar and neurobiological changes after several weeks of intermittent access to sugar (2). In these studies, rodents are, for example, food deprived for 12 h and then have access to lab chow or sugar for 12 h. These paradigms are often criticized for being artificial and, thus, to have low value for making inferences about a possible sugar addiction in humans.

However, I would argue that these paradigms match quite well to the eating styles of some individuals. For example, individuals with bulimia nervosa (BN) engage in binge eating, but undereat on non-binge meals (3, 4). That is, food intake may be restricted throughout the day, followed by a binge episode (which usually includes high-calorie, e.g., high-sugar, foods) in the evening. The same eating topography may be seen in other weight-concerned individuals who try to restrict their food intake [“restrained eaters” (5)], although not exhibiting full-blown binge episodes. To summarize, animal models are indeed an important part of the food addiction hypothesis and human studies are lacking to support some of the results found in those studies. Nevertheless, the paradigm of intermittent access to food may parallel eating topography of some individuals with restrained or disordered eating behavior.

## Evidence for Food Addiction Based on DSM-5

Secondly, it is argued that “there is very little evidence for food addiction” based on the DSM-5 criteria for substance use disorder (SUD). Most articles in which the concept of food addiction is discussed refer to the substance dependence criteria in DSM-IV. In 2013, the DSM-5 was published and diagnostic criteria for SUDs now include 4 additional symptoms [11 symptoms in total (6)].

To the best of my knowledge, only one study has examined the new DSM-5 criteria in relation to eating behavior yet. In that study (7), a semi-structured interview was conducted, responses of which were qualitatively analyzed. Results showed that obese participants with binge eating disorder (BED), and to a lesser extent also those without BED, met the full criteria for SUD. Although participants rarely met three of the four new criteria, most of them met the new criterion of *craving, or a strong desire or urge to use the substance*. Admittedly, findings of this study should not be overinterpreted as the validity of the semi-structured interview is questionable and sample size was small. Undoubtedly, future studies are urgently needed that examine if the new DSM-5 SUD criteria can be translated to eating behavior and if those criteria are met by individuals who engage in over eating or binge eating [for a more detailed discussion (see Meule and Gearhardt, submitted)]. However, dismissing the appropriateness of the new DSM-5 criteria with regard to food addiction in the first place seems unfair.

## Food Addiction Diagnoses in Different Weight Categories

Thirdly, the author suggests that “the Yale Food Addiction Scale [(YFAS) Ref. (8)] criteria may not be appropriate for diagnosing food ‘addiction”’ based on the fact that most obese people do not meet those criteria, but a substantial portion of underweight and normal-weight subjects do. Indeed, studies using this scale found prevalence rates of food addiction of about 5–10% in community or student samples and about 15–25% in obese samples (9, 10). In morbidly obese individuals or individuals with BED, prevalence rates range between approximately 30 and 50% (9, 10).

However, why do these findings disprove the validity of the YFAS? In my opinion, it rather shows that body mass is a poor measure when talking about food addiction. In most cases, obesity is the consequence of modest daily excess of energy consumption over energy expenditure (11). In fact, the error in caloric balance in obese persons is on average <0.0017% per year (12). Eating behavior in such individuals is certainly not comparable to an addiction but is rather related to eating styles such as *grazing* or *mindless eating*. Instead, addiction is much more comparable to binge eating such as in BED or BN (13, 14) and this is exactly what is found using the YFAS (15, Meule et al., submitted). To conclude, the notion that food addiction may be responsible for the high prevalence rates of obesity and that obesity by itself represents an addictive behavior is outdated (15, 16) and the YFAS has contributed to these insights. Instead, food addiction is rather related to binge eating behaviors and the YFAS – although it may not be perfect – appears to be a helpful assessment tool in this context.

## Food Addiction and Brain Imaging

Fourthly, another argument is that “brain imaging studies […] do not support an addiction model.” This is based on a critical review by Ziauddeen and colleagues (16), which was, in turn, controversially discussed (17–19). Specifically, the authors found that brain imaging studies that involved presentation of food-cues in obese individuals with or without BED are inconsistent. Although brain activations in such studies are often related to prefrontal, limbic, or paralimbic areas, involvement of specific areas differs across studies. Furthermore, although there are similarities in brain responses to food and drugs, substantial differences have also been noted (20).

Nonetheless, common substrates were identified in meta-analyses (21). Inconsistencies in brain imaging studies are partly driven by the heterogeneity of the samples studied. Preferably, future studies that investigate the food addiction model should include individuals who actually receive a food addiction diagnosis (e.g., using the YFAS) and a control group of individuals who do not receive a food addiction diagnosis. Thus, it may be unjustified to conclude that neuroimaging studies do not support a food addiction model, as many of the existing studies were not specifically tailored to investigate this.

## Necessity and Potential Downsides of the Food Addiction Model

Finally, the author concludes that “much of the food-related pathology that is seen clinically can be explained and treated without invoking addiction, and in some cases using an addiction model may lead to further food-related pathology.” The food addiction model surely involves the danger of possibly creating a new stigma (22, 23) or to move attention away from the individual’s responsibility in weight regulation such as engaging in physical activity (24, 25). Moreover, current psychological treatments of BED are actually quite successful (26) and, thus, may not need to be adapted according to a food addiction model.

However, it has also been found that the concept of food addiction has a more positive public perception compared with alcohol or tobacco use and that the food addict label may be less vulnerable to public stigma than other addictions (22, 23, 27). Moreover, case reports exist, which show that providing an addiction framework may be helpful for some individuals, for example, those struggling with overweight and diet failures (28, 29) or with eating disorders such as BN (30). Thus, the food addiction model may be beneficial in some instances and may not be necessary or has potential downsides in others. However, drawing straightforward conclusions is not possible yet.

## Conclusion

The idea that some forms of overeating may represent an addictive behavior and that specific foods may have an addiction potential has been discussed in the scientific literature for decades (31). In the 2000s, scientific interest on food addiction has strongly increased in the light of the obesity pandemic and the rise of neuroimaging studies (32). Unfortunately, “this argument resonates strongly with the media and the public and has been perpetuated rather uncritically” (1) (p. 5). I unequivocally agree with the author that (a) media reports do not appropriately address the controversial concept of food addiction, (b) many findings from animal studies are not yet replicated in human studies, (c) obesity does not represent an addiction by itself, (d) brain imaging studies are inconsistent, and that (e) the necessity or potential downside of the food addiction concept in treatment or public health issues is still unclear. However, those are issues that will likely be addressed in future studies. Thus, it would be unjustified to dismiss the concept of food addiction based on limited data (18).
